# High expression level of CXCL1/GROα is linked to advanced stage and worse survival in uterine cervical cancer and facilitates tumor cell malignant processes

**DOI:** 10.1186/s12885-022-09749-0

**Published:** 2022-06-28

**Authors:** Xiaxia Man, Xiaolin Yang, Zhentong Wei, Yuying Tan, Wanying Li, Hongjuan Jin, Baogang Wang

**Affiliations:** 1 Department of Oncologic Gynecology, The First Hospital of Jilin University, Changchun, Jilin People’s Republic of China; 2 Department of Geriatrics, The First hospital of Jilin University, Changchun, Jilin People’s Republic of China; 3 Department of Echocardiography, The First hospital of Jilin University, Changchun, Jilin People’s Republic of China; 4 Department of Plastic Surgery, The First Hospital of Jilin University, Changchun, Jilin 130021 People’s Republic of China; 5 Department of Cardiac Surgery, The First Hospital of Jilin University, Changchun, Jilin 130021 People’s Republic of China

**Keywords:** Uterine cervix cancer, CXCL1, Malignant behavior, Extracellular regulated protein kinases

## Abstract

**Background:**

CXCL1 belongs to a member of the ELR + CXC chemokine subgroups that also known as GRO-alpha. It has been recognized that several types of human cancers constitutively express CXCL1, which may serve as a crucial mediator involved in cancer development and metastasis via an autocrine and/or paracrine fashion. However, the expression pattern and clinical significance of CXCL1 in human uterine cervix cancer (UCC), as well as its roles and mechanisms in UCC tumor biology remains entirely unclear.

**Methods:**

The expression and clinical significance of CXCL1 in UCC tissues was explored using immunohistochemistry and bioinformatics analyses. The expression and effects of CXCL1 in HeLa UCC cells were assessed using ELISA, CCK-8 and transwell assays. Western blotting experiments were performed to evaluate the potential mechanism of CXCL1 on malignant behaviors of HeLa UCC cells.

**Results:**

The current study demonstrated that CXCL1 was expressed in HeLa UCC cells, PHM1-41 human immortalized cervical stromal cells, as well as cervical tissues, with UCC tissues having an evidently high level of CXCL1. This high level of CXCL1 in cancer tissues was notably related to poor clinical stages and worse survival probability, rather than tumor infiltration and patient age. In addition, CXCL1 expression was extremely correlated with CCL20, CXCL8 and CXCL3 cancer-associated chemokines expression. In vitro, the growth and migration abilities of HeLa cells were significantly enhanced in the presence of exogenous CXCL1. Gain-function assay revealed that CXCL1 overexpression significantly promoted growth and migration response in HeLa cells in both autocrine and paracrine manners. Finally, we found that CXCL1 overexpression in HeLa cells influenced the expression of ERK signal-related genes, and HeLa cell malignant behaviors derived from CXCL1 overexpression were further interrupted in the presence of the ERK1/2 blocker.

**Conclusion:**

Our findings demonstrate the potential roles of CXCL1 as a promoter and a novel understanding of the functional relationship between CXCL1 and the ERK signaling pathway in UCC.

**Supplementary Information:**

The online version contains supplementary material available at 10.1186/s12885-022-09749-0.

## Background

Uterine cervix cancer (UCC), a common malignancy, represents the fourth most frequently occurring cancer and the fourth leading cause of cancer-related death in females worldwide [1]. It is estimated that 57,000 cases of UCC were newly diagnosed in 2018, and 311,000 patients were overwhelmed by this disease [1]. To date, several etiologies, including human papillomavirus (HPV) infection, immuno-suppression (particularly HIV infection), alcohol consumption, smoking, parity, numerous sexual partners, and oral intake of contraceptives, have been identified as predominant risk factors of cervical cancer; however, persistent HPV infection is the most common cause. In fact, HPV infection markedly increases the incidence of UCC [2, 3]. The persistence of inflammation among women with HPV infection is responsible for malignant transformation of the cervical epithelium [3], suggesting that cancer-related inflammatory reaction is predominantly involved in UCC pathogenesis.

Chemokines or chemoattractant cytokines are categorized into four subfamilies, namely CXC, CC, C, and CX3C, based on the number and spacing of the conserved cysteine residues near the N-terminus. The essential roles of chemokines in biology are mediated through their combinatorial interaction with the G protein-coupled chemokine receptors [4]. Early studies on chemokines were primarily focused on their roles in infection or inflammatory responses. Chemokines may act as mediators in the recruitment of various leukocyte populations, including neutrophils, lymphocytes, monocytes, and eosinophils, to damaged tissues [5]. However, increasing evidence has revealed that various chemokines may also play a critical role in many tumor processes, including tumor growth, angiogenesis, metastasis, and neutrophil infiltration, through their roles in tumor-promoting inflammation response [6].

CXCL1, also known as GRO-alpha, is a member of the ELR + CXC chemokine subgroups characterized by the presence of a conserved glutamic acid-leucine-arginine (ELR) motif [7]. CXCL1-related functions are regulated through its binding to the CXCR2 receptor [8]. To date, increasing evidence has demonstrated that the expression levels of CXCL1 and CXCR2 are frequently upregulated in several human cancers [7, 9–11], and CXCL1 may serve as a source of autocrine/paracrine signal for the malignant process in the tumor microenvironment [12, 13]. For example, silencing CXCL1 resulted in a significant reduction in tumor proliferation through its effect on apoptosis induction in HCC, suggesting that a network of autocrine signals may be involved in CXCL1-associated tumor biology [14]. Tumor cells with a rather high CXCL1 level may also trigger the infiltration of tumor-associated macrophages (TAMs) and cancer-associated fibroblasts (CAF) into the tumor microenvironment. CXCL1 derived from TAMs/CAF can play a favorable role in regulating intercellular adhesion and the crosstalk among tumor cells and TAMs/CAF and further results in an increased tumor proliferation in a paracrine manner [15].

In this study, we aimed to determine the expression pattern of CXCL1 protein in a commercial uterine cervical tissue microarray, as well as to evaluate its relationship with clinical characteristics. We also explored the potential exogenous autocrine and paracrine mechanisms involved in CXCL1-mediated UCC tumor biology, possibly via mitogen activated protein kinases (MAPK)/extracellular signal-regulated kinase (ERK)-dependent signal pathway.

## Materials and methods

### Immunohistochemistry using tissue microarray

A commercial uterine cervical tissue microarray (Bioaitech Co., Ltd, China) with 40 cancer tissues and 40 non-cancerous tissues was used to evaluate CXCL1 protein expression. Cancer samples ranged in age from 22 to72 years while non-cancer samples ranged in age from 32 to 68 years. The cancer tissue samples were classified into 3 clinical stages and 3 pathological grades. The sample characteristics are shown in Table 1. Immunohistochemical staining was conducted, and protein expression was determined using a primary antibody targeting CXCL1 (1:100; cat. no. TY2074, Immunoway, USA); the primary antibody was further matched with the HRP-linked secondary antibody (Boster Biological Technology, China) at a dilution of 1:200. Immunoreactivity was graded according to the following parameters: staining intensity and the percentage of positive cells. Staining intensity was scored as 1 (weak), 2 (moderate), 3 (strong), and 4 (super strong), and the percentage of positive cells was scored as 1 (< 25%), 2 (25–50%), 3 (50–75%), and 4 (> 75%). The total score for CXCL1 staining was calculated as the sum of the scores of both parameters.

**Table 1:** Sample characteristics in a uterine cervix tissue microarray

Sample characteristic	UCC (case)	Non-cancers (case)
Age, year		
≤ 50	22(range from 22–50)	28 (range from 32–50)
> 50	18(range from 51–72)	12 (range from 51–68)
Clinical stage		
I	29	
II-III	11	
Pathological grade		
1	10	
2	15	
3	10	

### Bioinformatics analysis

UALCAN database (http://ualcan.path.uab.edu/) was employed to explored the association of CXCL1 or CXCR2 mRNA expression with patient survival. To further elucidate the positive genes that associated with CXCL1 signal in UCC, we further explored the correlation between CXCL1 and other genes expression using this database.

### Cell lines, cell culture, and cell transfection

HeLa human cervical cancer cells and PHM1-41 human immortalized cervical stromal cells were obtained from American type culture collection (ATCC, USA). HeLa and PHM1-41 cells were respectively cultured in RPMI‑1640 and Dulbecco’s Modified Eagle’s Medium (DMEM; Gibco, USA) supplemented with 10% fetal bovine serum (FBS; Gibco, USA) containing antibiotics (100 units/ml penicillin and 100 mg/ml streptomycin) in a 37 °C humidified incubator with 5% CO_2_. To stably overexpress the CXCL1 gene in HeLa and PHM1-41 cells, the full-length cDNA of human CXCL1 was subcloned into the pcDNA3.1 vectors, and the specific vector targeting cells were transfected using the Lipofectamine 3000 kit (Invitrogen, USA), according to the manufacturer’s instructions. Finally, the transfection efficiency of CXCL1 in HeLa and PHM1-41 cells was assessed using ELISA.

### ELISA analysis

The culture supernatant derived from cell medium was collected and the concentration of the CXCL1 secretory protein was determined using Human CXCL1 ELISA Kit (Boster Biological Technology, China) following the manufacturer’s instruction. Briefly, after culture for 24 h, cell culture media were collected. 100 μL of culture supernatant and different concentrations of recombinant CXCL1 standard protein were added to each well of the plate. The plate was then incubated at 37 °C for 90 min. Following triple rinsing with washing buffer, the plate was coated with 100 μL biotin-conjugated primary antibody against CXCL1 prior to incubation at 37 °C for 60 min. After three rounds of rinsing, the plate was coated with 100 μL of working fluid and further incubated at 37 °C for 30 min. Upon formation of the immunocomplex, the plate was washed five times, and 100 μL of TMB developer solution was placed into each well for colorimetric determination. After the reaction at 37 °C for 20 min, the absorbance of each well was immediately measured at 450 nm using a spectrophotometer (BioTek Instruments, USA). The level of the CXCL1 protein in the culture supernatant was quantified and analyzed based on a standard curve constructed using the absorbance values of the CXCL1 standard protein.

### Cell proliferation detection

Three separate experiments were used to evaluate HeLa cell viability. (I) Exogenous effect: HeLa cells at a density of 2 × 10^3^ cells per well were seeded into each well of 96-well plates supplemented with 100 µL medium containing various concentrations of human recombinant CXCL1 (PeproTech, USA). (II) Autocrine effect: The same number of HeLa cells overexpressing CXCL1 and their mock controls, as described in experiment I, were grown in 96-well plates. One hundred microliter of medium supplemented with or without the ERK1/2 inhibitor, PD98059 (Sigma, USA), was added to each well. (III) Paracrine effect: First, the conditioned medium (CM) was prepared as follows: CXCL1-overexpressing PHM1-41 cells and their mock controls were inoculated in 100-mm diameter dishes at a density of 5 × 10^6^ cells per 1000 µL of medium. After incubation at 37 ˚C for 24 h, the culture supernatant was harvested by centrifugation, and CM from PHM1-41 cells was produced by adding different proportions of the supernatant into complete medium. In the paracrine experiment, HeLa cells at a density of 2 × 10^3^ cells per well were seeded into 96-well plates, and each well was incubated with 100 µL of different proportions of CM. The cells in the above three groups were incubated at 37 ˚C for 24 h, 48 h, and 72 h, and cell viability was determined using the Cell Counting Kit-8 (CCK-8) assay.

### Cell migration assay

Consistent with the proliferation assay, the following three experiments were carried out for the migration assay. (I) Exogenous effect: The same number of HeLa cells at a density of 2 × 10^3^ cells/well were seeded at the top chamber in 100 μL serum-free medium. The low chamber was coated with 600 μL serum-supplemented medium containing various concentrations of human recombinant CXCL1. (II) Autocrine effect: CXCL1-overexpressing HeLa cells (2 × 10^4^) and their mock controls in 100 μL serum-free medium containing the ERK inhibitor, PD98059, or no inhibitor were seeded at the top chamber, whereas 600 μL medium with 10% FBS was placed at the low chamber for chemoattraction. (III) Paracrine effect: HeLa cells were maintained in the upper chamber as described in experiment I, and 600 μL medium containing various proportions of CM supplemented with 10% FBS was placed in the low chamber. Twenty-four hours after migration, the cells attached to the bottom surface of the Transwell filters were fixed and stained with methanol/crystal violet solution.

### Western blot analysis

Total cell lysates were prepared from cell pellets using RIPA buffer (Solarbio, China) following the manufacturer’s protocol. After quantification of protein concentration, 40 µg of protein extracts was subjected to western blotting analysis. Different primary antibodies, including anti-p-ERK (1:1,000 dilution; ImmunoWay, USA), anti- ERK (1:1,000 dilution; ImmunoWay, USA), anti-cyclin D1 (1:5,000 dilution; Abcam, USA), anti-Bax (1:1,000; OriGene, China), and anti-β‑actin (1:1,000 dilution; Boster, China), were utilized in the analysis. Thereafter, the samples were incubated with various HRP-conjugated secondary antibodies (Boster Biological Technology, China). Immune complexes were visualized using the Enhanced Chemiluminescence (ECL) Kit (Thermo Fisher Scientific, USA), and band intensities were calculated using the Gel imaging system (Tanon, China).

### Statistical analysis

All assays were carried out at least three times and SPSS 20.0 software was applied for statistical analyses. All values are expressed as the mean value and standard deviation. Statistical difference between two groups was determined using analysis of variance followed by the Student’s t-test. Statistically significance was defined as a *p*-value < 0.05.

## Results

### CXCL1 is highly expressed in UCC tissues

**Fig. 1:**
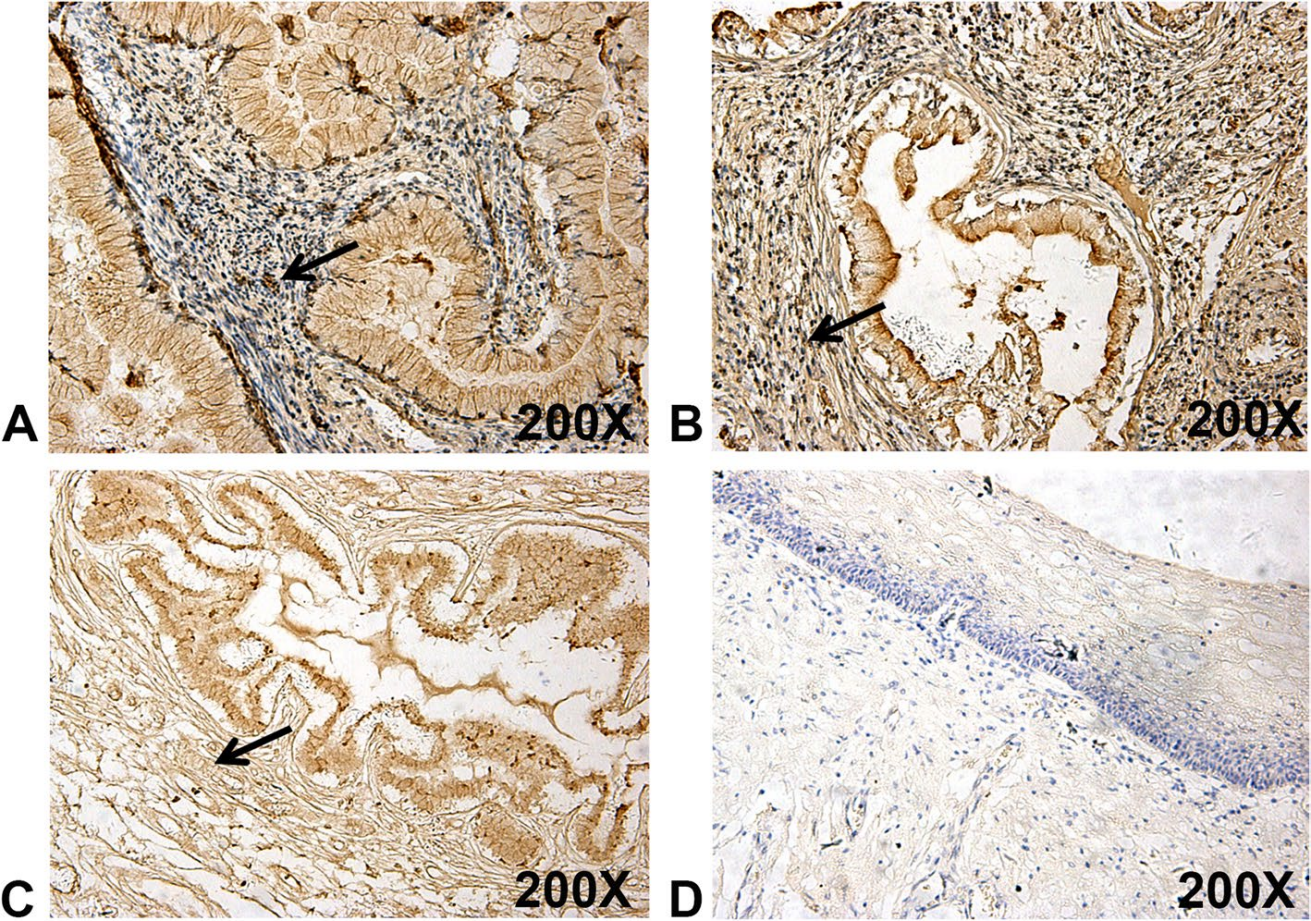
CXCL1 is highly expressed in tissues with UCC. Strong immunohistochemical staining of CXCL1 in tissues with stage I (**A**), II (**B**) and III (**C**), comparing non-cancerous tissues with weak staining (**D**), was detected in a uterine cervical tissue microarray. Arrows, CXCL1 expression in stroma

To assess the clinical significance of CXCL1 in UCC, CXCL1 immunoreactivity was explored in uterine cervical tissue microarray. The results from the immunohistochemistry assay demonstrated that CXCL1 protein expression level was surprisingly increased in UCC tissues compared to non-cancerous tissues (Fig. 1A-D). Of the UCC tissues, it was further demonstrated that CXCL1 upregulation was correlated with poor clinical stages due to the increased identification of CXCL1 in stage II-III UCC tissues. Herein, a close relationship was not found between CXCL1 expression and other clinical characteristics, including tumor infiltration and patient age (Table 2). In addition, our immunohistochemistry also demonstrated that CXCL1 was expressed in stroma (Arrows, Fig. 1), suggesting that CXCL1 derived from stromal cells may be involved in the progression of UCC.

**Table 2:** Immunostaning intensities of CXCL1 protein in a uterine cervix tissue microarray

Sample characteristic	Subgroup	Score(‾χ ± S)	*P* value
Non-cancer	–-	5.40 ± 1.57	0.0304
UCC	–-	6.10 ± 1.26	
Clinical stage	I	5.83 ± 1.31	0.0240
	II-III	6.82 ± 0.75	
Pathological grade	1	6.10 ± 1.37	0.5765
	2	6.47 ± 0.83	
	3	6.30 ± 0.82	
Age(years)	≤ 50	6.25 ± 1.29	0.3620
	> 50	5.88 ± 1.20	

### Elevated expression of CXCL1 was associated with worse patient survival in CESC

**Fig. 2:**
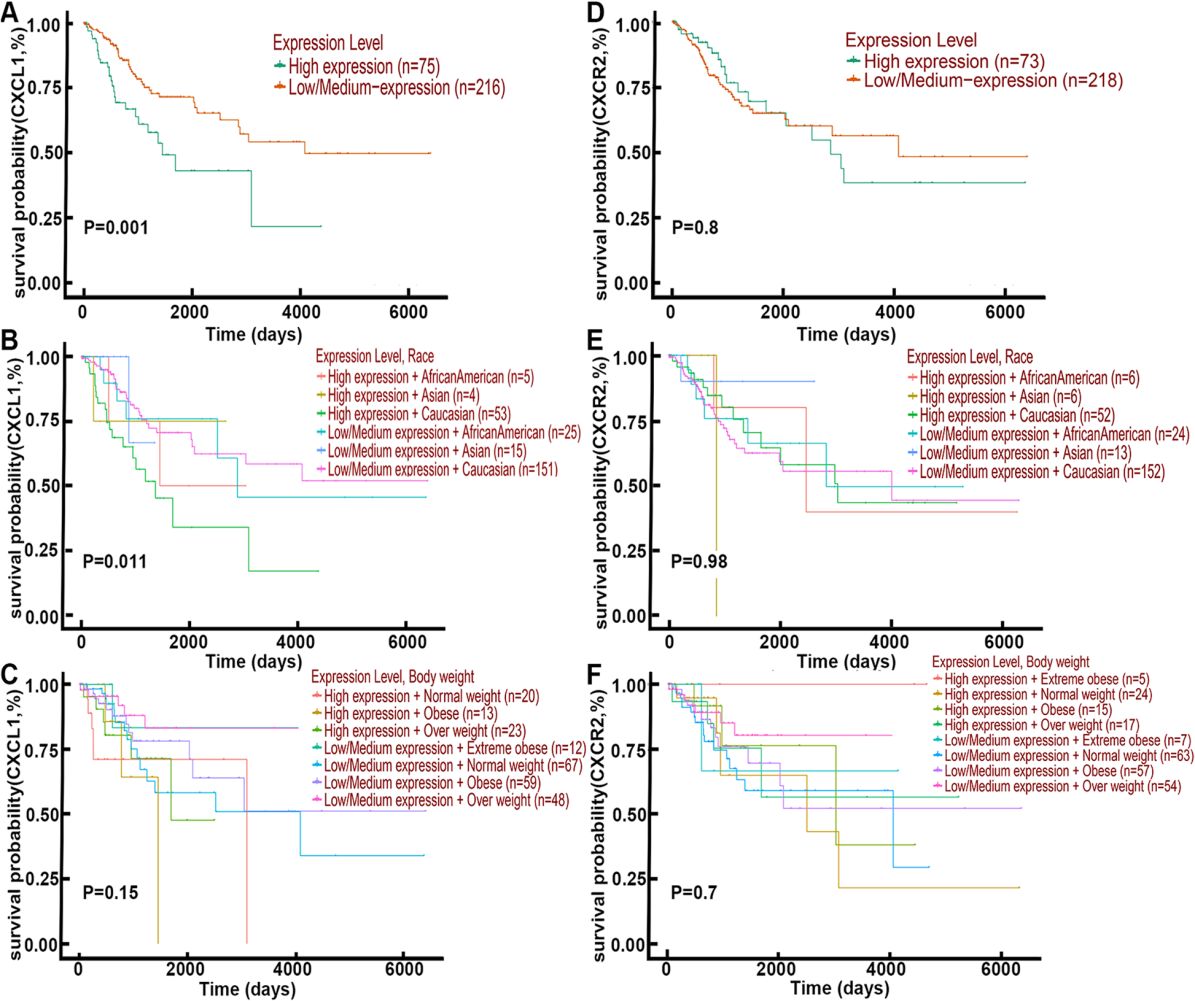
High expression of CXCL1 positively correlated with worse survival in patients with CESC. **A** The role of CXCL1 expression level on patient overall survival probability. **B** The role of CXCL1 expression level and race on patient overall survival probability. **C** The role of CXCL1 expression level and body weight on patient overall survival probability. **D** The role of CXCR2 expression level on patient overall survival probability. **E** The role of CXCR2 expression level and race on patient overall survival probability. **F** The role of CXCR2 expression level and body weight on patient overall survival probability

Cervical cancer contains squamous cell carcinoma, adenocarcinoma, adenosquamous carcinoma, and other rare tumors, in which cervical squamous cell carcinoma (CESC) is the most common type of malignancy. We then used bioinformatic methods to study the CESC samples. The findings regarding the association between CXCL1 expression and patient survival probability are shown in Fig. 2. Survival curves on dataset showed patients with high-level expression of CXCL1 have a trend towards a worse overall survival probability compared with those with those with low-level expression of CXCL1 (Fig. 2A). Further studies indicated that CXCL1 expression on survival probability was significantly associated with race of patient, although only 5 africa/american and 4 asia samples with high expression of CXCL1 were involved in the study (Fig. 2B), However, high CXCL1 expression was not associated with body weight (Fig. 2C). In addition, bioinformatics analysis also showed that CXCR2 was expressed in CESC samples, but its expression was not related to patient survival (Fig. 2D-F).

### CXCL1 expression was positively correlated with cancer-associated chemokines expression

**Fig. 3:**
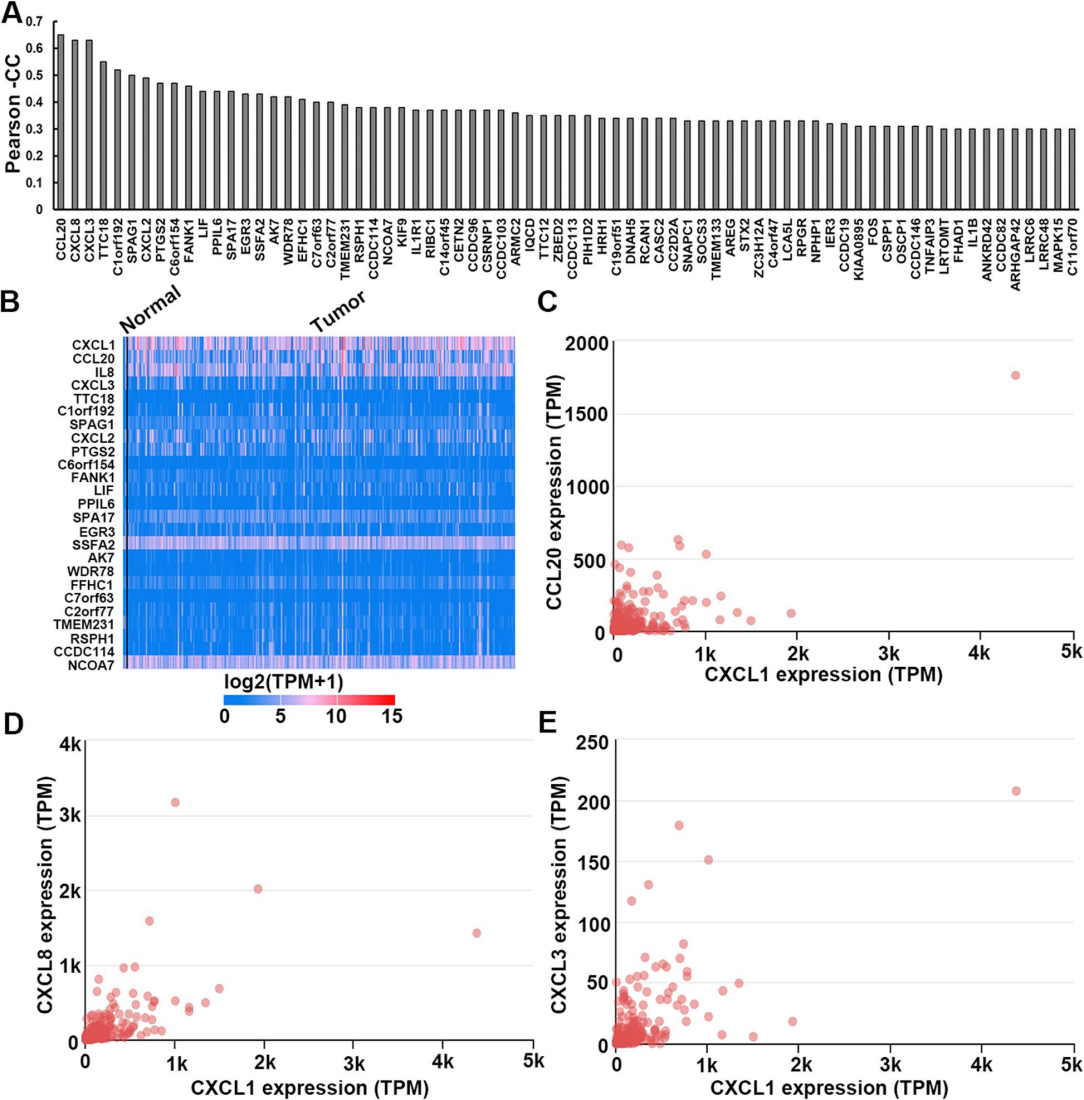
CXCL1 expression was positively related with cancer-associated chemokines expression in CESC. **A** The genes that positively correlated with CXCL1 expression. **B** Heatmap of expression associated assay of 25 most relevant genes. **C** Gene expression correlation assays between CXCL and CCL20 (**C**), CXCL8 (**D**) or CXCL3 (**E**)

Gene expression associated assay was used to identify the genes that were positively correlated with CXCL1 expression in CESC, and the genes with extremely low expression with median TPM (transcripts per milliom) < 0.5 were filtered out of the results. A total of 73 genes with pearson correlation coefficient (PCC) ≥ 0.30 were found (Fig. 3A and B), in which cancer-associated chemokines CCL20, CXCL8 and CXCL3 were extremely associated with CXCL1 expression (Fig. 3C, D and E).

### Exogenous CXCL1 contributes to the growth, and migration of UCC cells

**Fig. 4:**
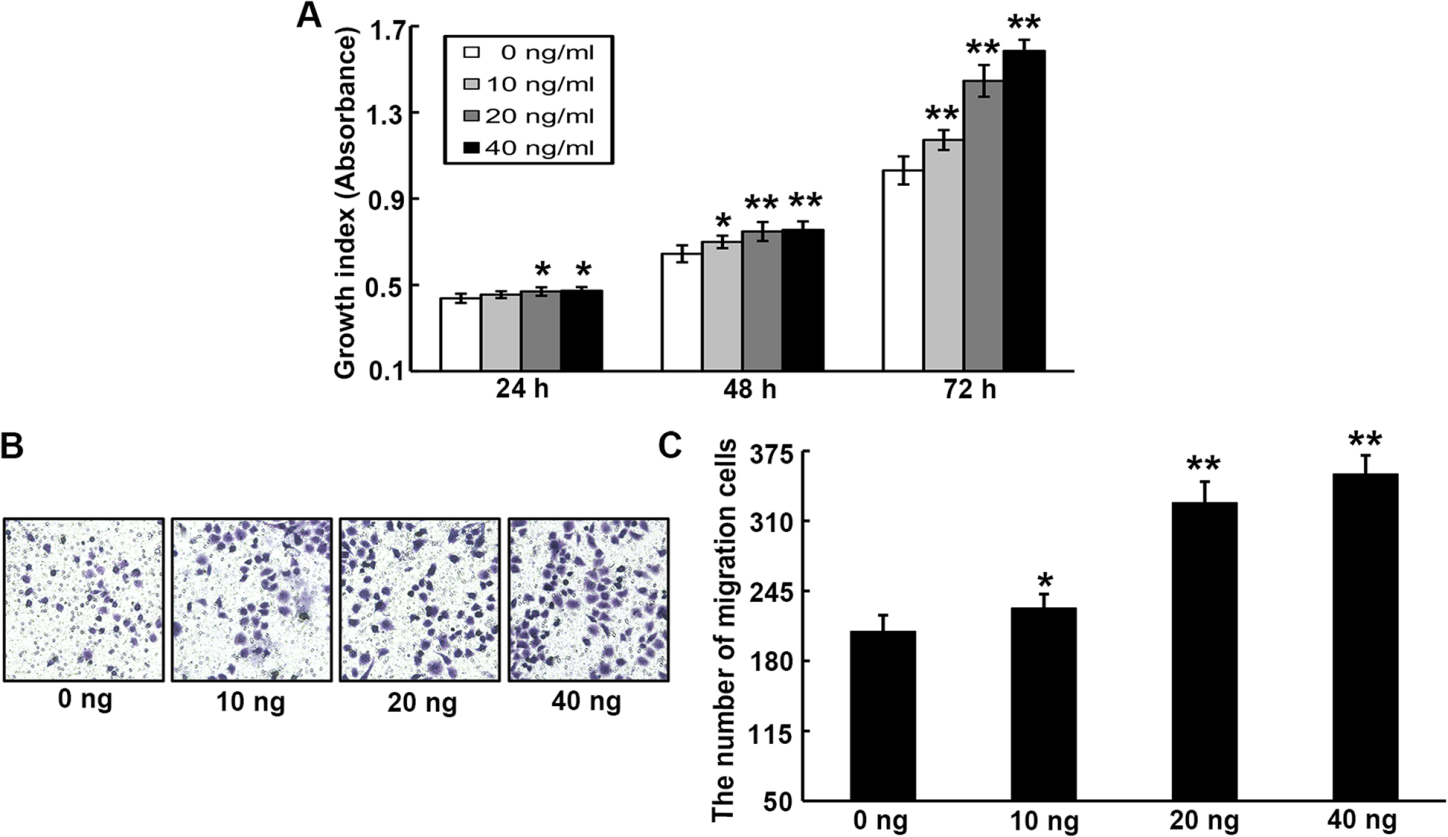
Exogenous CXCL1 facilitates the malignant behaviors of HeLa cells. **A** The role of different concentrations of exogenous CXCL1 on the proliferation of HeLa cells was tested by CCK-8 assay. **B-C** Cell migration ability was determined by transwell analysis after HeLa cells treatment with different concentrations of exogenous CXCL1. *P < 0.05, **P < 0.01

To determine the functional roles of CXCL1 in UCC, exogenous CXCL1 was first employed as a mediator to investigate its potential for the growth and migration,of HeLa cells. After 24, 48, and 72 h incubation, the CCK-8 assay revealed that exposure to exogenous CXCL1 (10, 20, or 40 ng/ml) induced an evident dose-dependent increase in HeLa cell growth ability (Fig. 4A). Accordingly, exogenous administration of CXCL1 at concentrations of 10, 20, or 40 ng/ml also evidently enhanced the migration ability of HeLa cells (Fig. 4B and C).

### CXCL1 overexpression enhances malignant phenotypes of UCC cells via an autocrine manner

**Fig. 5:**
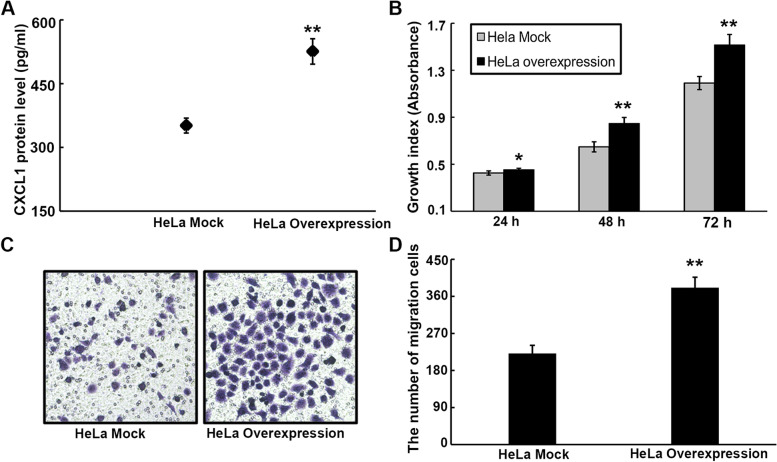
CXCL1 overexpression contributes to proliferation, migration and apoptosis of HeLa cells via a autocrine manner. **A** The protein expression of CXCL1 in the supernatant derived from HeLa cells overexpressing CXCL1 and their mock controls medium was assessed by ELISA assay. **B** The proliferation ability of HeLa cells overexpressing CXCL1 was significantly increased as compared to their mock controls. **C-D** The cell migration capacity was notably enhanced after CXCL1 overexpression in HeLa cells. *P < 0.05, **P < 0.01

Because CXCL1 was expressed in uterine cervical tisssues, we intended to explore its expression in HeLa cells. Based on the results of ELISA, the CXCL1 protein was detected in the HeLa cell culture supernatant (Fig. 5A), suggesting that HeLa cells express endogenous CXCL1, which might be further transported into the extracellular fluid as a secretory protein. To determine whether the autocrine mechanism is involved in CXCL1-mediated malignant processes in UCC, HeLa cells overexpressing CXCL1 were constructed via gene transfection. The results showed that the secretory CXCL1 protein was prominently elevated in CXCL1-overexpressing cells relative to their mock controls (Fig. 5A), indicating that stable transfected HeLa cells with CXCL1 expression were constructed as expected. Additionally, the CCK-8 and transwell assays demonstrated that CXCL1 overexpression in HeLa cells resulted in an evident increase in cell growth (Fig. 5B) and migration (Fig. 5C and D).

### Stromal cell-derived CXCL1 facilitates UCC cell malignant processes in a paracrine manner

**Fig. 6:**
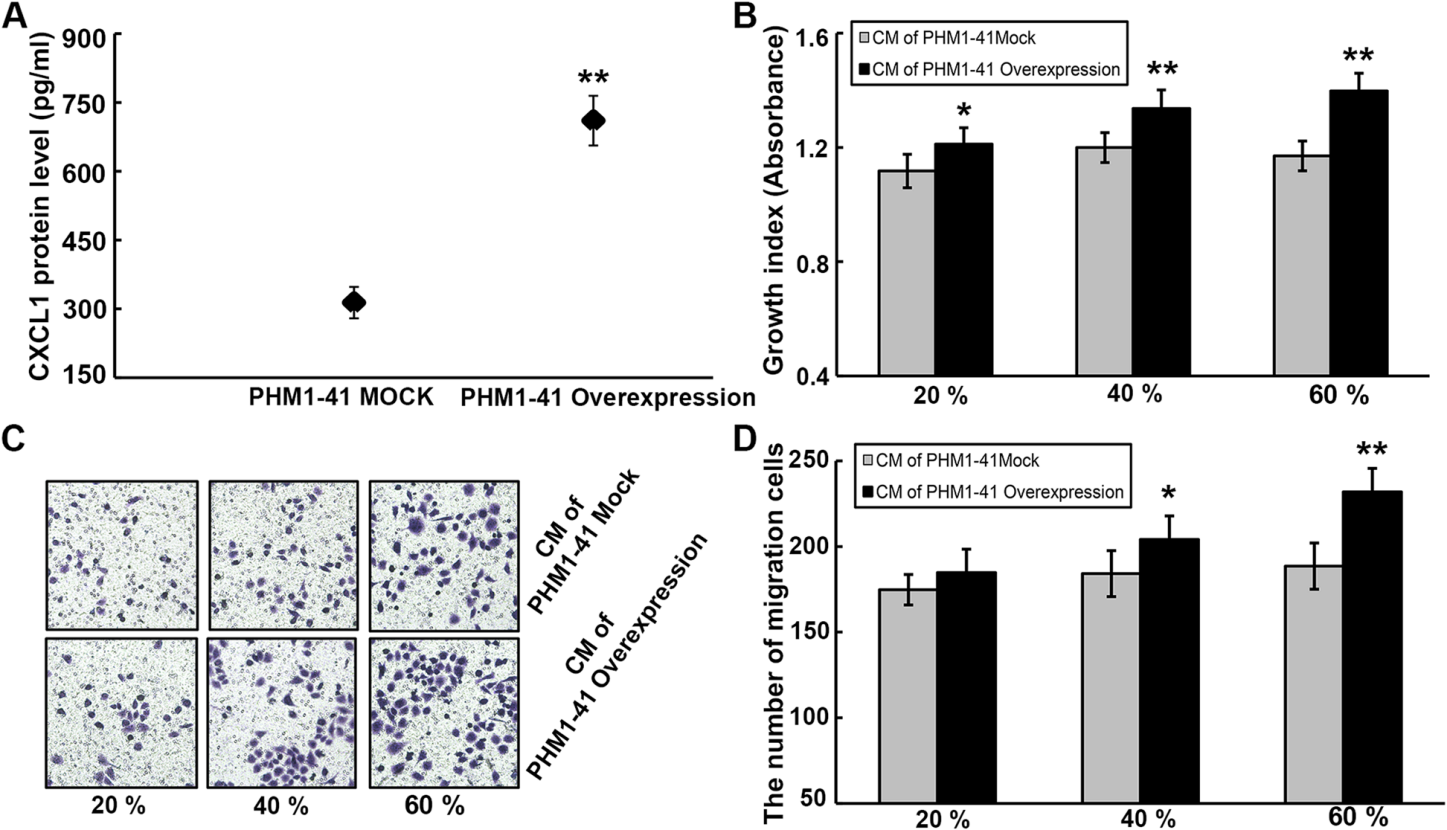
Stromal cell-derived CXCL1 enhances oncogenic potential of HeLa cells via a paracrine manner. **A** CXCL1 protein level in PHM1-41 cells overexpressing CXCL1 and their mock controls-derived supernatant was determined using ELISA assay. **B** The growth ability of HeLa cells treatment with CM from PHM1-41 cells overexpressing CXCL1 and their mock controls was detected by CCK-8 assay. **C-D** The effect of various proportions of CM derived from PHM1-41 cells overexpressing CXCL1 and their mock controls on the HeLa cell migration was explored by transwell analysis. *P < 0.05, **P < 0.01

To gain further insights into the potential paracrine mechanism that involve CXCL1, PHM1-41 cells overexpressing CXCL1 and their mock controls were established. After the verification of transfection efficiency by ELISA (Fig. 6A), CM was prepared by mixing the supernatant from PHM1-41 cell medium with complete medium containing 10% FBS in various proportions. According to the CCK-8 and transwell assays, treating HeLa cells with CM derived from CXCL1-overexpressing PHM1-41 cells at different proportions evidently enhanced the cell growth (Fig. 6B) and migration (Fig. 6C and D) abilities in a proportion-dependent manner.

### ERK signal regulates UCC cell malignant processes through its direct effect on the survival-associated gene

**Fig. 7:**
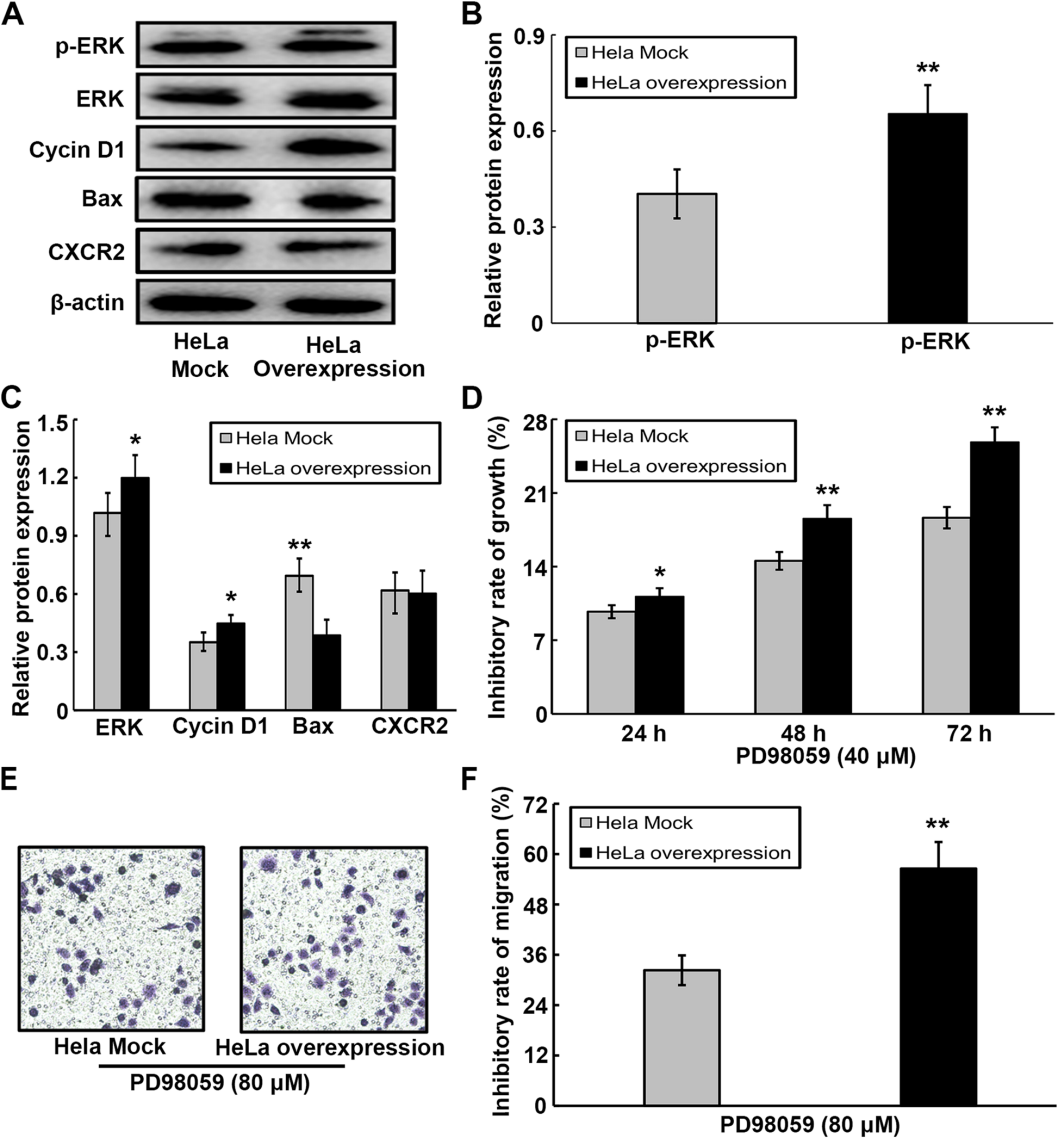
ERK signal involves in CXCL1-mediated malignant phenotypes in HeLa cells. **A** Expression of ERK, p-ERK, Cyclin D1 and Bax at protein levels from HeLa cells overexpressing CXCL1 and their mock controls were assessed by western blotting. **B** The protein level of the p-ERK was quantitated and normalized to total ERK from western blotting. **C** Protein expression of ERK, Cyclin D1 and BAX were statistically analyzed from western blotting, and their bands were normalized to β-actin. **D** The proliferation inhibition rate of ERK inhibitor PD98059 on HeLa cells overexpressing CXCL1 and their mock controls was determined by CCK-8 assay. **E–F** The effect of ERK inhibitor PD98059 on migration inhibition rate of HeLa cells overexpressing CXCL1 and their mock controls was assessed by transwell analysis. *P < 0.05, **P < 0.01

Increasing evidence has indicated that numerous chemokines are critical for tumor-specific processes as they activate several signaling pathways, especially MAPK/ERK. Therefore, in the current study, the roles of CXCL1 in regulating the expression of ERK signal-related genes were explored. Based on western blotting, a predominant increase in the expression levels of ERK, pERK, and cyclin D1, and a marked decrease in the expression of Bax were detected in CXCL1-overexpressing HeLa cells compared to their mock controls (Fig. 7A-C). Our results also indicated that the CXCL1 receptor CXCR2 was constitutively expressed on Hela cells, but overexpression of CXCL1 in Hela cells did not affect the expression of CXCR2 (Fig. 7A, C). To determine the underlying mechanism whereby the ERK signal regulates the roles of CXCL1 in UCC, we further assessed the malignant behaviors of HeLa cells in the presence and absence of the ERK inhibitor, PD98059; the rates of proliferation and migration inhibition are expressed as follows: inhibition rate (%) = (the index of cell treatment without PD98059- the index of cell treatment with PD98059) / (the index of cell treatment without PD98059) × 100%. Our results showed that the growth (Fig. 7D) and migration (Fig. 7E and F) abilities of CXCL1-overexpressing HeLa cells were evidently reduced to significant levels compared to their mock controls in the presence of the ERK inhibitor, PD98059. Such finding suggests the capacity of PD98059 to interrupt CXCL1-mediated UCC tumor processes.

## Discussion

Human CXCL1 is a member of the ELR + CXC chemokines, which contain epithelial neutrophil-activating protein-78 (ENA-78/CXCL5), granulocyte chemotactic peptide-2 (GCP-2/CXCL6), neutrophil-activating peptide-2 (NAP-2/CXCL7), interleukin-8 (IL-8/CXCL8), and growth-related oncogene (GRO) chemokines, that have three homologs (GRO α, β, and γ), also defined as CXCL1, CXCL2, and CXCL3, respectively [16]. The biological activities of ELR + CXC chemokines are primarily mediated by their interaction with the same receptor, namely CXCR2 [17]. Recently, several members of ELR + CXC chemokines have been shown to be constitutively expressed in various types of human cancers, and their overexpression may be implicated in multiple mechanisms of cancer progression, including tumor cell proliferation, survival, migration, invasion and tumor angiogenesis [18–23]. Studies on the mechanisms revealed that the involvement of ELR + CXC chemokines in tumor progression is dependent on the activation of several signal pathways, such as p38 MAPK/ERK, TAK1/NFκB, and Snail/E-cadherin [24–26].

In the current study, we demonstrated that the expression level of CXCL1 in UCC tissues was markedly elevated, and its higher expression was extremely associated with poor clinical stages. Consistently, it has been suggested that CXCL1 expression was frequently upregulated in several human tumors, such as melanoma, and breast and bladder cancers, and increased expression pattern of CXCL1 was predominantly relevant to several clinicopathological features, including high grade, advanced stage, and positive invasion in bladder cancer in vivo [27–30].

Many studies have highlighted the crucial roles of CXCL1 in cancer development and progression. For example, secreted CXCL1 or CXCL1 overexpression in bladder cancer and melanoma was revealed to be correlated with the enhanced proliferative and metastatic properties of cancer cells, and CXCL1 in vitro was sufficient for cancer cells to generate tumors in a nude mouse xenograft model [27, 30]. To evaluate the potential roles of CXCL1 in UCC biology, we employed recombinant CXCL1, CXCL1-overexpressing HeLa, and PHM1-41 cells to derive the effects of CXCL1 on HeLa tumor cell malignant behaviors. Our data revealed that CXCL1 recombinants and CXCL1 overexpression effectively promoted the cell proliferation and migration properties in both exogenous and autocrine manners.

Generally, it is agreed that the tumor microenvironment is composed of cancer cells, extracellular matrix, and different types of stromal cell subsets, which contain fibroblasts, vessel cells (composed of endothelial cells, smooth muscle cells, and pericytes), and diverse inflammatory leukocytes (including neutrophils, macrophages, dendritic cells, mast cells, and lymphocytes) [31]. To date, people have gained a deeper understanding of the crosstalk among these cell types, which contributes to the formation of a tumor-specific microenvironment and further facilitates the oncogenic process via a paracrine loop involving the interaction of chemokines with their corresponding receptors [15]. In thyroid cancer, tumor cell-derived soluble factors facilitated in vitro mast cell activation and recruitment, and the mast cell-producing mediators, especially CXCL1, most effectively enhanced the growth, survival, and invasion of cancer cells [32]. In addition, in breast cancer, CXCL1 can attract CD11b^+^Gr1^+^ myeloid stromal cell population to the tumor microenvironment, which in turn generates several chemokine members that can serve as paracrine stimulators for the survival of tumor cells [13]. In the present study, we employed gene transfection to establish CXCL1-overexpressing PHM1-41 cervical stromal cells, which can secrete a significantly high level of CXCL1 into the medium. Further, the supernatants derived from the medium of CXCL1 overexpressing PHM1-41 cell can behave as a promoting mediator for the growth and migration of HeLa cells, indicating that CXCL1-paracrine interaction plays a crucial role in tumor-associated malignant behaviors. In addition, our previous laboratory findings revealed the critical CXCL3-paracrine mechanism where supernatants derived from stromal cells overexpressing CXCL3 increased HeLa cell growth and migration abilities [33].

In the current study, we finally revealed that CXCL1 is a crucial chemokine that enhances UCC development by regulating extracellular signal-regulated kinase (ERK) signal-related genes, including ERK, p-ERK, cyclin D1, and Bax. ERK signaling is an essential transduction pathway that has received considerable attention because of its important effects in the regulation of various physiological and pathological processes. The activation of the ERK signaling pathway may stimulate tumor cell proliferation through its ability to initiate the expression of crucial growth-stimulating genes, such as cyclin D1 and early growth response-1 (EGR-1) [34, 35]. The CXCL1/CXCR2 axis has been shown to participate in the regulation of ERK1/2 phosphorylation in esophageal cancer, which is responsible for the transcription of the EGR-1 gene, the crosstalk of among CXCL1/CXCR2 axis, ERK1/2 and EGR-1 result in a rapid increase in tumor cell proliferation [36]. Besides its impact on cell proliferation, ERK signaling is also closely involved in regulating the expression of many other genes responsible for anti-apoptosis, angiogenesis, and metastasis. In the current study, we confirmed that the exogenous administration and overexpression of CXCL1 can effectively promote the proliferation and migration of HeLa cells, and result in a increase in cyclin D1 gene expression, as well as a decrease in Bax gene expression. Cyclin D1 is a key regulator of cell cycle progression associated with occurrence, differentiation, invasiveness and prognosis of various human malignancies [37]. Cyclin D1 may be involved in the crosstalk between tumor cell and stromal components in tumor microenvironment, it has been shown that overexpression of cyclin D1 in stromal fibroblasts promotes its transformation into a tumor associated fibroblasts phenotype, and stromal cyclin D1 exhibits a prominent ability to induce secretion of proinflammatory cytokines including CXCL1 from stromal fibroblasts, and further facilitate tumor cell proliferation, and suppress tumor cell apoptosis [38]. Apoptosis or programmed cell death (PCD) is a controlled form of cell death that allows the body to precisely control the number of cells in tissues, and is mainly regulated by the pro- and anti-apoptotic BCL-2 family members, with Bax as one of the most important mediators for the induction of the apoptotic process [39]. It has been increasingly recognized that apoptosis is frequently inhibited in many cancer types, which is at least associated with the low expression of pro-apoptotic genes, such as Bax, Bak, Bad, and Bcl-Xs. Such findings indicate that the disruption of the balance between pro- and anti-apoptosis is critical for numerous human tumor processes [39].

## Conclusion

In summary, the current study demonstrated that CXCL1 expression was increased in tissues with UCC, and its higher expression was predominantly correlated with poor clinical stages. In vitro, CXCL1 may contribute the UCC cell malignant behaviors through exogenous, autocrine and paracrine manners, suggesting CXCL1 may be a potential diagnostic marker or therapeutic target in UCC.

## Supplementary Information

**Additional file 1. **

## Data Availability

The data applied in the bioinformatics analysis were obtained from UALCAN open-access database (http://ualcan.path.uab.edu/) mentioned in the materials and methods section.

## Abbreviations

UCC: Uterine cervix cancer
CXCL1: C-X-C motif chemokine 1
HPV: Human papillomavirus
TAMs: Tumor-associated macrophages
CAF: Cancer-associated fibroblasts
ERK: Extracellular regulated protein kinase
EGR-1: Early growth response-1
Bax: Bcl-2 Associated X Protein
MAPK: Mitogen activated protein kinases
