# Supplementary figures and images for: High expression level of CXCL1/GROα is linked to advanced stage and worse survival in uterine cervical cancer and facilitates tumor cell malignant processes
Source: BMC Cancer. 2022 Jun 28;22:712. doi: 10.1186/s12885-022-09749-0 (PMC9241244; doi:10.1186/s12885-022-09749-0)

p-ERK


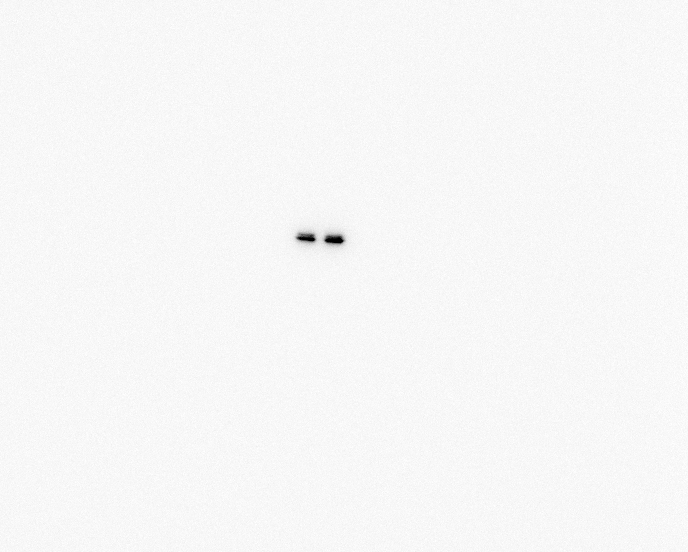


ERK


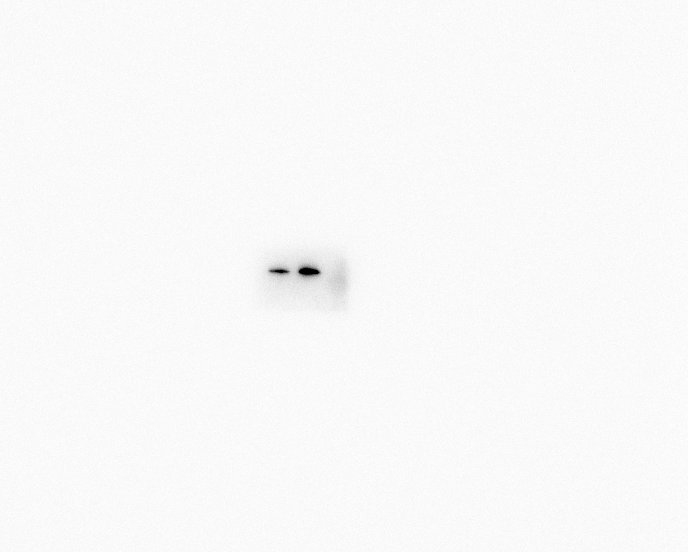


Cycline D1


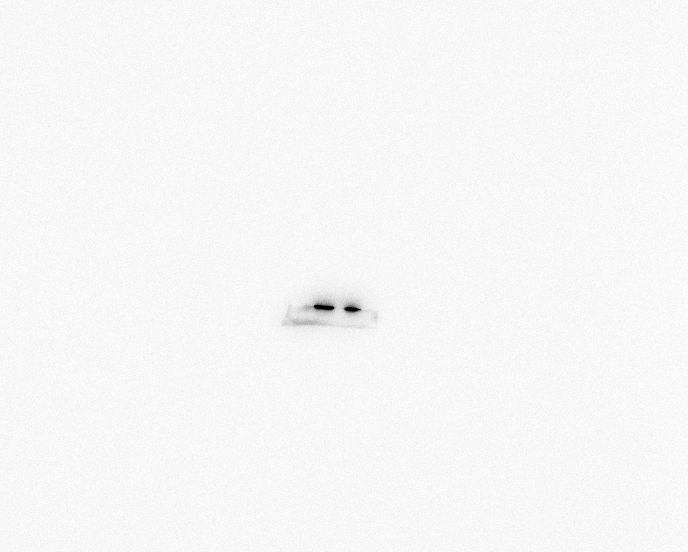


BAX


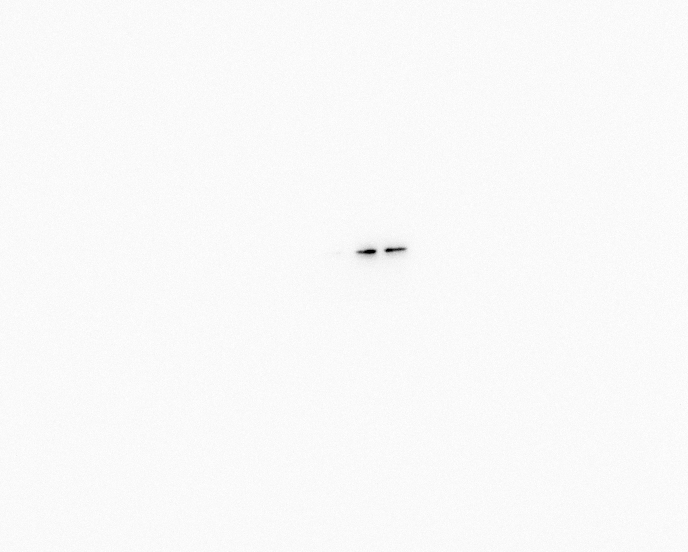


CXCR2


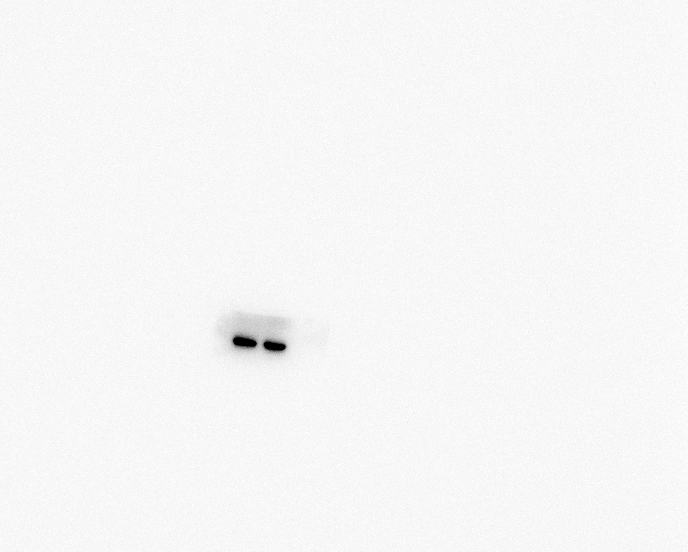


actin

Supplement: Supplementary file 1 — Additional file 1. [file 12885_2022_9749_MOESM1_ESM.docx]
